# Comedication and Polypharmacy With ADHD Medications in Adults: A Swedish Nationwide Study

**DOI:** 10.1177/1087054720923725

**Published:** 2020-06-01

**Authors:** Le Zhang, Andreas Reif, Ebba Du Rietz, Tyra Lagerberg, Agnieszka Butwicka, Brian M. D’Onofrio, Kristina Johnell, Nancy L. Pedersen, Henrik Larsson, Zheng Chang

**Affiliations:** 1 Karolinska Institutet, Stockholm, Sweden; 2 Goethe University Frankfurt, Germany; 3 Medical University of Warsaw, Poland; 4 Stockholm County Council, Sweden; 5 Indiana University, Bloomington, USA; 6 Örebro University, Sweden

**Keywords:** ADHD medications, comedication, somatic medications, polypharmacy

## Abstract

**Objective:** Evidence regarding comedication among individuals with ADHD is lacking, especially in adults. This study investigated comedication and polypharmacy with ADHD medications in adults. **Method:** We identified adults dispensed with ADHD medications during 2013 in Sweden and matched them to controls. Logistic regression was used to calculate odds ratios (ORs) of receiving other medications. **Results:** Individuals receiving ADHD medications had higher risk of receiving any major classes of somatic medications (ORs ranged from 4.1, 95% confidence interval [CI] = [4.0, 4.3], to 7.4, 95% CI = [6.5, 8.5] across age groups). They were more likely to receive respiratory system, alimentary tract and metabolic system, and cardiovascular system medications. In addition, they had higher risk of receiving any other psychotropic medications. The proportion of polypharmacy with five or more medication classes increased from 10.1% to 60.4% from 18 to 64 years. **Conclusion:** Comedication was more common in adults receiving ADHD medications. Potential benefits and harms of comedication and polypharmacy require further research. *(J. of Att. Dis. XXXX; XX*[*X*] *XX-XX)*

## Introduction

ADHD is a common neurodevelopmental disorder, affecting 5% to 8% of children (Polanczyk et al., 2014; Thomas et al., 2015) and 3% of adults worldwide (Simon et al., 2009). Although ADHD usually has its onset in childhood, follow-up studies have shown that the disorder often persists into adulthood (Faraone et al., 2006) and that it is associated with long-term health problems (Ingram et al., 1999; Nigg, 2013). The prevalence of ADHD medication use among adults has increased substantially over the last decade. For example, the prevalence of any ADHD medication use in 2010 was 1.42% in North America and 0.47% in northern Europe, with annual average increase of 10% and 19% from 2000, respectively (Raman et al., 2018).

It is well established that patients with ADHD have high rates of psychiatric comorbidities (Comer et al., 2010; Karlstad et al., 2016; Larson et al., 2011). Emerging evidence has shown that young individuals with ADHD often have somatic comorbidities, such as metabolic syndrome (Fluegge & Fluegge, 2018), disorders of chronic inflammation (e.g., asthma and allergic rhinitis; Cortese et al., 2018; Schmitt et al., 2016), and cardiovascular disorders (Arruda et al., 2010), but much less is known about comorbidities among middle-aged and older adults with ADHD. As both adult ADHD and the aforementioned comorbid disorders feature a chronic, long-lasting course, it may result in long treatment periods with simultaneous use of multiple medications. Although use of multiple medications has been associated with increased risks of adverse drug events, including nonadherence, cumulative toxicity, and drug–drug interactions (Sarkar, 2017; Sirois et al., 2016), very little is known about comedication and polypharmacy patterns associated with ADHD medication. Moreover, there is limited information on how to address comedication in treatment guidelines for ADHD (National Institute for Health and Care Excellence [NICE], 2018; Swedish National Board of Health and Welfare [Swedish: Socialstyrelsen], 2014; Wolraich et al., 2019).

By taking advantage of Swedish national registers, this nationwide study investigated the comedication (including both somatic [nonpsychotropic] and psychotropic medications) and polypharmacy patterns with ADHD medications among adults.

## Method

### National Registers and Study Population

Data were available through linkage of the Prescribed Drug Register (PDR), the Total Population Register (TPR), and the National Patient Register (NPR) in Sweden based on the unique personal identification numbers (Ludvigsson et al., 2009). The PDR covers information on all prescribed medications dispensed at pharmacies in Sweden since July 2005, which includes drug identity (defined using Anatomical Therapeutic Chemical [ATC] codes), dose, dates of dispensed prescriptions, and the prescriber’s profession and practice (Wettermark et al., 2007). The proportion of invalid dispensation entries in the PDR is expected to be below 2% (Furu et al., 2010). The TPR was established by Statistics Sweden and covers demographic information since 1968. This includes information on births, deaths, place of residence, civil status, migration, relations, and citizenship of all Swedish residents, and has virtually complete coverage of births and deaths (Ludvigsson et al., 2016). The NPR contains data on inpatient diagnoses since 1973 (Ludvigsson et al., 2011) and outpatient diagnoses since 2001 based on the International Classification of Diseases (ICD). Information from the PDR was used to identify dispensations of ADHD medications and other medications. The TPR was used to obtain information on date of birth and sex. Information from the NPR was used to identify individuals with ADHD diagnosis.

The target population included all individuals aged 18 to 64 years who were residing in Sweden during 2013. Among whom, all individuals who had at least one dispensed prescription of ADHD medication during the study period were identified. A randomly selected 1:1 control group of individuals without any ADHD medication prescriptions in 2013 was created by matching on year of birth and sex as individuals with ADHD medication prescriptions.

### Measures

Information on ADHD medication was defined from the PDR. Four stimulants (methylphenidate [ATC code: N06BA04], amfetamine [N06BA01], dexamfetamine [N06BA02], and lisdexamfetamine [N06BA12]) and two nonstimulants (atomoxetine [N06BA09] and guanfacine [C02AC02]) were included in this study. For sensitivity analysis, information on ADHD diagnosis was obtained from the NPR, where diagnostic codes in ICD-9 (1987–1996, codes 314) and ICD-10 (1997–2013, codes F90) were used.

To describe prescription patterns, we retrieved information on the source of prescription (primary care, nonpsychiatric specialist care, and psychiatric care) and the duration of prescriptions from the PDR. In accordance with previous studies (Lagerberg et al., 2019; Lichtenstein et al., 2012), an individual was considered to be receiving treatment during the interval between two dispensed ADHD medication prescriptions if these occurred less than 6 months apart. The longest treatment period for each individual during a year was considered.

Comedication with ADHD medication was defined as dispensed prescriptions of any other medication within 6 months before and after an ADHD medication prescription (Lagerberg et al., 2019). Medications were ascertained based on first-level ATC codes, which include alimentary tract and metabolism (gastrointestinal tract and metabolic system (A); cardiovascular system (C); dermatologicals (D); genitourinary system and reproductive hormones (G); systemic hormonal preparations (H); anti-infectives (antibiotics, antifungals, antiparasitic agents, and antivirals) for systemic use (J); antineoplastic and immunomodulating agents (L); musculoskeletal system (M); nervous system (N); antiparasitic products, insecticides, and repellents (P); respiratory system (R); and sensory organs (S). Detailed information on ATC codes of medications included in our study is shown in Supplemental Table S1.

In addition to these main drug classes, we investigated dispensation of other psychotropic medications among individuals receiving ADHD medications, including antipsychotics (N05A); anxiolytics, hypnotics, and sedatives (N05B or N05C); antidepressants (N06A); antiepileptic drugs (N03A); drugs used in addictive disorders (substance use disorders, N07B); and opioid pain medications (N02A; Supplemental Table S1).

### Statistical Analyses

Patterns of ADHD medication prescriptions in relation to ADHD medication type, source of prescription, and duration of prescription in 2013 are reported as number and percentage. We examined the proportion of individuals receiving any somatic and psychotropic medications among individuals receiving ADHD medications and controls, as well as for each major ATC class. Logistic regression models were performed to estimate odds ratios (ORs) for receiving each drug class in those receiving ADHD medications versus controls, with adjustment for sex and age as a categorical variable (Pearce, 2016). ORs and 95% confidence intervals (CIs) were presented by age group (young adults [18–29 years], middle-aged adults [30–49 years], older adults [50–64 years]). Polypharmacy pattern (measured as the number of major classes of medications in addition to ADHD medications) was presented for individuals receiving ADHD medications and controls. All statistical analyses were performed using SAS version 9.4 (SAS Institute) and we used a threshold of *p* < .05 for statistical significance. Figures were created in R software version 3.5.0.

## Results

We identified 41,840 adults aged 18 to 64 years residing in Sweden who dispensed at least one prescription of ADHD medications in 2013, including 20,629 young adults (men 11,220 [54.39%]), 16,889 middle-aged adults (men 8,931 [52.88%]), and 4,322 older adults (men 2,339 [54.12%]). Table 1 shows the prescription patterns of ADHD medication in young, middle-aged, and older adults in 2013. Overall, methylphenidate was the most commonly dispensed medication (82%–90% in all age groups), whereas the proportion of lisdexamfetamine and guanfacine accounted for less than 1%. Psychiatric care was the most prevalent source of prescription (ranged from 87.90%–92.84%), followed by other specialist care (from 6.31%–10.50%) and primary care (from 0.86%–1.36%). The majority of individuals who dispensed ADHD prescriptions experienced treatment periods longer than 1 year for all age groups. The prescription patterns were similar between men and women (Supplemental Table S2).

**Table 1.** Patterns of ADHD Medication Prescriptions Among Adults in 2013, Stratified by Age Group.

Characteristics	Young adults^a^ (*N* = 20,629)	Middle-aged adults (*N* = 16,889)	Older adults (*N* = 4,322)
Type (%)			
Methylphenidate	18,381 (89.10%)	14,672 (86.87%)	3,584 (82.92%)
Atomoxetine	3,236 (15.69%)	2,232 (13.22%)	547 (12.66%)
Lisdexamfetamine	137 (0.66%)	177 (1.05%)	33 (0.76%)
Amfetamine	141 (0.68%)	337 (2.00%)	218 (5.04%)
Dexamfetamine	442 (2.14%)	945 (5.60%)	254 (5.88%)
Guanfacine	7 (0.03%)	1 (0.01%)	1 (0.02%)
Source (%)			
Primary care	280 (1.36%)	145 (0.86%)	69 (1.60%)
Specialist care^b^	1,393 (6.75%)	1,065 (6.31%)	454 (10.50%)
Psychiatric care	18,956 (91.89%)	15,679 (92.84%)	3,799 (87.90%)
Duration of medication (%)			
Single prescription^c^	1,438 (6.97%)	686 (4.06%)	197 (4.56%)
Short term (≤6 months)	2,189 (10.61%)	1,164 (6.89%)	273 (6.32%)
Medium term (6–12 months)	2,434 (11.80%)	1,424 (8.43%)	310 (7.17%)
Long term (>12 months)	14,568 (70.62%)	13,615 (80.61%)	3,542 (81.95%)

a Young adults refer to patients aged 18–29 years; middle-aged adults refer to patients aged 30–49 years; older adults referred to patients aged 50–64 years.

b Specialist care excluding psychiatry.

c Single prescription here entails single dispensed prescriptions of any ADHD medication. Switching between different types of ADHD medications is not captured by this number.

### Comedication With Somatic Medications in Individuals Receiving ADHD Medications

Overall, the percentage of individuals coprescribed with any somatic medications increased with age, from 76.7% in young adults to 93.4% in older adults (Table 2). The proportion of dispensation with any somatic medication was higher among individuals receiving ADHD medications than controls, with ORs ranging from 4.1 (95% CI = [4.0, 4.3]) in young adults to 7.4 (95% CI = [6.5, 8.5]) in older adults.

**Table 2.** Dispensations of Any Somatic and Other Psychotropic Medications Among Individuals Receiving ADHD Medications.

Comedication	Age group	ADHD drug users (*N* = 41,840)	Control group (*N* = 41,840)	Odds ratio [95% CI]
Somatic medications	Young adults (18–29 years)	15,818 (76.7%)	10,134 (49.1%)	4.1 [4.0, 4.3]
	Middle-aged adults (30–49 years)	14,631 (86.6%)	8,951 (53.0%)	6.2 [5.9, 6.6]
	Older adults (50–64 years)	4,037 (93.4%)	2,865 (66.3%)	7.4 [6.5, 8.5]
Psychotropic medications	Young adults (18–29 years)	13,812 (67.0%)	2,673 (13.0%)	15.5 [14.7, 16.3]
	Middle-aged adults (30–49 years)	14,458 (85.6%)	3,846 (22.8%)	21.7 [20.5, 23.0]
	Older adults (50–64 years)	3,859 (89.3%)	1,394 (32.3%)	18.6 [16.5, 20.9]

*Note.* CI = confidence interval.

**Figure 1.**
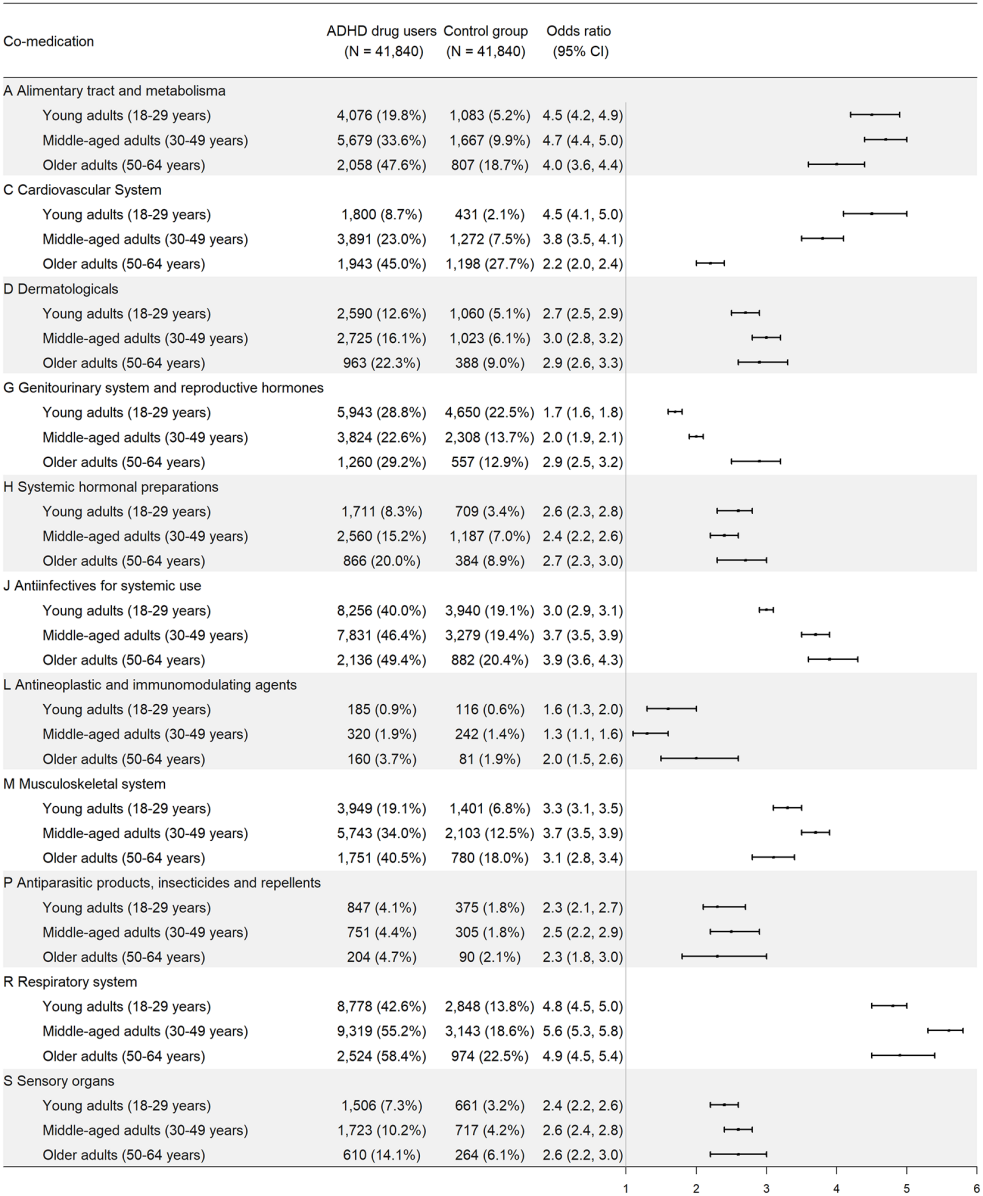
Dispensation of somatic medications (according to ATC classes) among individuals receiving ADHD medications, by age group. *Note.* ATC = Anatomical Therapeutic Chemical.

For each specific class of somatic medications, individuals receiving ADHD medications had higher odds of receiving them compared with controls for all age groups (Figure 1). Among all major classes of medications, respiratory system medications have the highest odds of being dispensed when comparing individuals with ADHD medication prescriptions with controls. The ORs ranged from 4.8 (95% CI = [4.5, 5.0]) among young adults to 5.6 (95% CI = [5.3, 5.8]) among middle-aged adults. Supplemental Table S3 lists the 10 most commonly dispensed medications in the respiratory system medication class among individuals receiving ADHD medications.

Alimentary tract and metabolic system medications are associated with the second highest ORs of being dispensed, in individuals with ADHD medication prescriptions versus controls. The ORs ranged from 4.0 (95% CI = [3.6, 4.4]) among older adults to 4.7 (95% CI = [4.4, 5.0]) among middle-aged adults. Supplemental Table S4 lists the 10 most commonly dispensed medications in this class among individuals dispensed with ADHD medications.

Cardiovascular system medications have the third highest ORs of being dispensed when comparing individuals with ADHD medication prescriptions with controls. The OR of receiving cardiovascular system medications was the highest among young adults (OR = 4.5, 95% CI = [4.1, 5.0]). The association attenuated with age, but remained significant among older adults (OR = 2.2, 95% CI = [2.0, 2.4]). Supplemental Table S5 lists the 10 most commonly dispensed medications in this class among individuals dispensed with ADHD medications.

Sensitivity analyses showed that the comedication patterns among individuals with both ADHD diagnosis and ADHD medication versus controls are similar to the main analysis (Supplemental Figure S1). Men and women receiving ADHD medications showed similar comedication patterns except that men had a lower proportion but higher OR of receiving genitourinary and reproductive hormone medications (Supplemental Figure S2).

### Comedication With Other Psychotropic Medications in Individuals Receiving ADHD Medications

The odds of comedication with any other psychotropic medications was substantially higher among individuals dispensed with ADHD medications than controls (Table 2), with highest ORs in middle-aged adults (OR = 21.7, 95% CI = [20.5, 23.0]), followed by older adults (OR = 18.6, 95% CI = [16.5, 20.9]) and young adults (OR = 15.5, 95% CI = [14.7, 16.3]).

**Figure 2.**
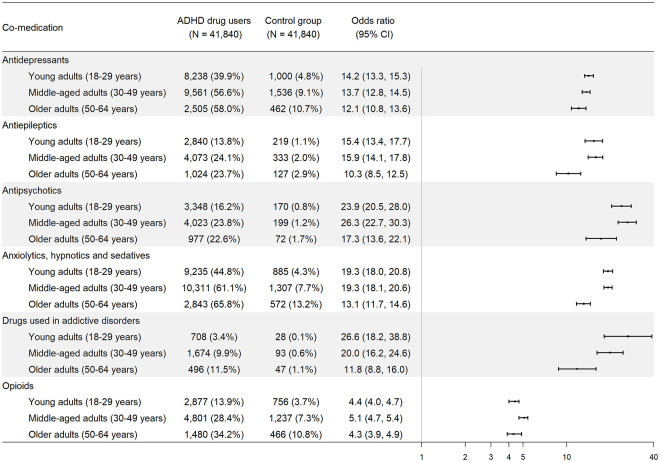
Dispensation of common psychotropic medications among individuals receiving ADHD medications, by age group.

Figure 2 shows the proportions and ORs for receiving each type of psychotropic medications in individuals receiving ADHD medication prescriptions versus controls. Overall, individuals with ADHD medication prescriptions were more likely to take all types of psychotropic medications than population controls. In young adults, medications used in addictive disorders had the highest OR of being dispensed (OR = 26.6, 95% CI = [18.2, 38.8]), whereas in middle-aged adults and older adults, comedication with antipsychotics was the highest, with ORs of 26.3 (95% CI = [22.7, 30.3]) and 17.3 (95% CI = [13.6, 22.1]), respectively. The results remained consistent in individuals with both ADHD diagnosis and ADHD medication (Supplemental Figure S3). Supplemental Tables S6 to S11 list the most commonly dispensed medications in each type of psychotropic medications among individuals receiving ADHD medications.

### Polypharmacy With ADHD Medications

**Figure 3.**
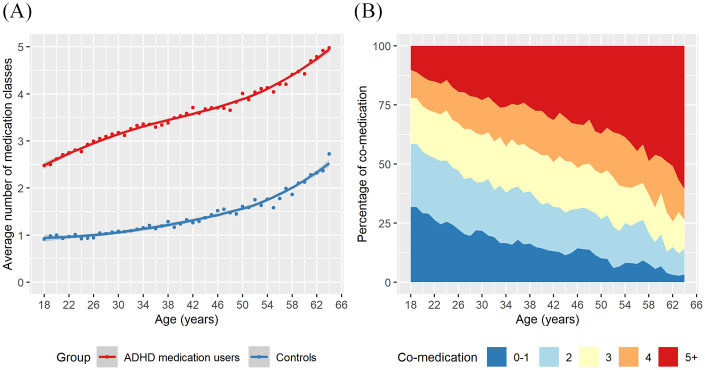
Comedication and polypharmacy among individuals receiving ADHD medications across age: (A) degree of polypharmacy, (B) number of comedicated drug classes.

Compared with population controls, individuals receiving ADHD medications had higher degree of polypharmacy from young adulthood to older age. They received on average 2.5 classes of other medications at age 18 to 5.0 classes at age 64, with the corresponding figures of 0.9 to 2.7 among population controls (Figure 3A). The proportion of polypharmacy with five or more medication classes increased from 10.1% at age 18 to 60.4% at age 64 among individuals receiving ADHD medications (Figure 3B).

## Discussion

This Swedish nationwide study provides a comprehensive overview of comedication and polypharmacy patterns associated with ADHD medication in adults. Comedication with somatic medications was more common in adults who received ADHD medications compared with the general population, in particular with respiratory medications, alimentary tract and metabolic system medications, and cardiovascular system medications. Comedication with psychotropic medications was also common. Individuals receiving ADHD medications presented high degree of polypharmacy across age.

We found that the most commonly comedicated respiratory medications among individuals receiving ADHD medications were alimemazine, mometasone, and terbutaline (Supplemental Table S3). These medications are mainly used for allergic reactions and asthma. The increased odds of receiving respiratory medications is in line with findings that ADHD is associated with chronic conditions, such as eczema (Buske-Kirschbaum et al., 2013), asthma (Cortese et al., 2018), and allergic rhinitis (Schmitt et al., 2016), and smoking behavior (Modesto-Lowe et al., 2010). Another possible explanation for the coprescription pattern is that some antihistamines (alimemazine, promethazine [which is actually a low-potency antipsychotic], and hydroxyzine) might be prescribed to alleviate anxiety disorders, sleep disorders, nausea and vomiting, and as unspecific sedative medicine.

The most commonly dispensed alimentary tract and metabolic system medications across all age groups included proton pump inhibitors omeprazole and esomeprazole (Supplemental Table S4). These medications are indicated for gastric/duodenal ulcers and gastroesophageal reflux disease (GERD). However, no studies to date have evaluated the potential link between ADHD and gastric/duodenal ulcers or GERD. It is possible that proton pump inhibitors could shorten the time to maximum plasma concentration of ADHD medications, though the evidence is scarce and there are variations between different types of ADHD medications (Haffey et al., 2009). Another possible explanation is that proton pump inhibitors may have beneficial effects on sleep problems, though there is conflicting evidence for this (Rassameehiran et al., 2016). Our study also found increased odds of coprescription of sodium fluoride and its combinations, which are both indicated for dental caries. There is little evidence on dental caries among ADHD in the existing literature, with only a few small sample size studies conducted with inconsistent findings (Manoharan & Krishnamoorthy, 2016). The association between ADHD and dental diseases warrants further investigation. In addition, insulin preparations and metformin had high odds of coprescription, which is in line with studies showing that ADHD is associated with both obesity and diabetes (Instanes et al., 2018; Nigg, 2013). Although there is research suggesting that methylphenidate is associated with reducing energy intake in obese patients (Leddy et al., 2004), there are no clinical practice guidelines in Sweden supporting the use of ADHD medications in the treatment of obesity.

Regarding cardiovascular system medications, the most commonly dispensed ones—propranolol, metoprolol, and enalapril—are indicated for hypertension and arrhythmias (Supplemental Table S5). This might be explained by cardiovascular events or symptoms associated with ADHD (Fuemmeler et al., 2011), although there is a lack of consistent evidence. A review has found that ADHD is not associated with cardiovascular diseases (Instanes et al., 2018), but this study was based on only a limited number of studies with subjective measurements. Another possible explanation is that the use of ADHD medications might be associated with cardiovascular symptoms (e.g., heart rate and blood pressure elevations; Mick et al., 2013). The largest study evaluating the effect of ADHD medications on serious cardiovascular events in adults showed no significant association (Habel et al., 2011), whereas another study found the opposite (Schelleman et al., 2012). Further studies are needed to ascertain the underlying mechanism of the observed association between ADHD medications and cardiovascular medications. Apart from that, although the ORs of cardiovascular system medication use attenuated in older adults, the absolute difference was substantial (45% in ADHD medication group vs. 27.7% in control group), suggesting that comedication of cardiovascular medications and ADHD medications should not be overlooked in older adults.

The high proportion of psychotropic medications among individuals dispensed with ADHD medications presented in our study is consistent with previous studies in adolescents (Comer et al., 2010). The highest ORs with drugs for addictive disorders and antipsychotics are in line with the well-established link between ADHD and substance use disorder and schizophrenia–spectrum disorders (Larsson et al., 2013; Skoglund et al., 2015). Shared genetic and environmental factors may play a role in the comorbidities among major psychiatric disorders (Pettersson et al., 2016). In addition, the proportion of adults receiving opioids, specifically codeine combinations, tramadol, and oxycodone, was high (Supplemental Table S11). The high rate of opioids prescription is an important public health concern in the United States and many European countries (Bosetti et al., 2019; Curtis et al., 2019; Tuminello et al., 2019). Our findings suggest individuals with ADHD medications warrant further attention, considering their vulnerability to substance abuse.

Our results suggest that ADHD patients face serious multimorbidity and higher degree of polypharmacy from young ages, which would put challenges for the management of ADHD, as well as other comorbid conditions (Vanderwal, 2016). Future studies, including pharmacoepidemiological studies using real-world data (Chang et al., 2019), are needed to study comedication of specific drug–drug pairs (Johnell & Klarin, 2007), especially those with potential drug–drug interactions as reported in clinical databases such as DynaMed or Lexicomp.

The results of this study should be considered in light of its limitations. First, we used dispensed prescriptions from the PDR when assessing medication utilization, but cannot verify whether the dispensed medications were consumed. Second, we did not have information on the underlying indications for dispensations of medications, which means that we were unable to determine whether any of the ADHD or comorbid medications were dispensed for other problems or off-label use. However, there is no indication other than ADHD for which the use of ADHD medications is licensed in Sweden, though off-label use of ADHD medications to alleviate symptoms caused by narcolepsy and multiple sclerosis is possible but rare (Zetterqvist et al., 2013). In addition, in the sensitivity analysis where we have explored the associations among individuals with ADHD diagnosis and matched controls, we found consistent results with the main analysis. Third, individuals receiving ADHD medications may be more likely to be prescribed with other medications due to the more frequent visits and exposure to medical environment compared with controls. Fourth, our study includes most of the medications in the ATC classification system but not all ATC codes. Fifth, our results on comedication do not imply a causal relationship between ADHD medications and excess use of additional medications. Sixth, we used data on medication dispensations in 2013. Further replication with more recent data is warranted, especially after the introduction of the American Psychiatric Association’s (2013) *Diagnostic and Statistical Manual of Mental Disorders* (5th ed.; *DSM-5*), which relaxed the age limit of symptom onset from age 7 to 12 years in diagnosis of ADHD (Epstein & Loren, 2013). Finally, the clinical practices on prescribing medications vary between countries (NICE, 2018; Swedish: Socialstyrelsen, 2014; Wolraich et al., 2019). Thus, the comedication patterns derived from Sweden may not generalize to other contexts.

### Clinical Implications

We found elevated rates of comedication and polypharmacy with somatic and psychotropic medications in adults receiving ADHD medications. Our findings provide new insights into the complex nature of comedication and polypharmacy with somatic medications among adults receiving ADHD medications, and call for future research to inform guideline development on optimal clinical practice.

## Supplemental Material

Supplementary_material – Supplemental material for Comedication and Polypharmacy With ADHD Medications in Adults: A Swedish Nationwide Study Click here for additional data file. Supplemental material, Supplementary_material for Comedication and Polypharmacy With ADHD Medications in Adults: A Swedish Nationwide Study by Le Zhang, Andreas Reif, Ebba Du Rietz, Tyra Lagerberg, Agnieszka Butwicka, Brian M. D’Onofrio, Kristina Johnell, Nancy L. Pedersen, Henrik Larsson and Zheng Chang in Journal of Attention Disorders
