# Supplementary material for: Comedication and Polypharmacy With ADHD Medications in Adults: A Swedish Nationwide Study
Source: J Atten Disord. 2020 Jun 1;25(11):1519–28. doi: 10.1177/1087054720923725 (PMC8369907; doi:10.1177/1087054720923725)

Table S1. Medications prescribed in Sweden, classified according to the Anatomic Therapeutic Chemical (ATC) Classification System.

First level of ATC codes		Second Level of ATC codes	Example
A	Alimentary tract and metabolisma	A01: Stomatological preparations	Sodium Fluoride
		A02: Drugs for acid-related disorders	Omeprazole
		A07: Antidiarrheals, intestinal antiinflammatory/anti-infective agents	Loperamide
		A10: Drugs used in diabetes	Metformin
		A03–A06, A08–A09, A11–A14, A16†; A15‡	
B	Blood and bloodforming organs	B01–B03, B05, B06†	
C	Cardiovascular system	C01: Cardiac therapy	Epinephrine
		C02: Antihypertensives	Clonidine
		C03: Diuretics	Furosemide
		C04: Peripheral vasodilators	Ergoloid mesylates
		C05: Vasoprotectives	Prednisolone
		C07: β-blocking agents	Propranolol
		C08: Calcium channel blockers	Amlodipine
		C09: Agents acting on the renin-agiotensin system	Enalapril
		C10: Lipid-modifying agents	Simvastatin
D	Dermatologicals	D04: Antipruritics, including antihistamines, anesthetics	Diphenhydramine
		D05: Antipsoriatics	Acitretin
		D06: Antibiotics and chemotherapeutics for dermatologic use	Demeclocycline
		D07: Corticosteroids, dermatologic preparations	Hydrocortisone
		D11: Other dermatologic preparations	Glycopyrronium
		D01–D03, D08–D10†	
G	Genitourinary system and reproductive hormones	G02: Other gynecological drugs	Methylergometrine
		G03: Sex hormones and modulators of the genital system	Estradiol
		G04: Urological drugs	Oxybutynin
		G01†	
H	Systemic hormonal preparations, excluding reproductive hormones and insulins	H01: Pituitary and hypothalamic hormones and analogs	Somatropin
		H02: Corticosteroids for systemic use	Hydrocortisone
		H03: Thyroid therapy	Thiamazole
		H04: Pancreatic hormones	Glucagon
		H05: Calcium homeostasis	Paricalcitol
J	Antiinfectives for systemic use	J01: Antibacterials for systemic use	Doxycycline
		J02: Antimycotics for systemic use	Fluconazole
		J04: Antimycobacterials	Rifampicin
		J05: Antivirals for systemic use	Valaciclovir
		J06–J07†	
L	Antineoplastic and immunomodulating agents	L01: Antineoplastic agents	Bendamustine
		L02: Endocrine therapy	Triptorelin
		L03: Immunostimulants	Filgrastim
		L04: Immunosuppressants	Mycophenolic acid
M	Musculoskeletal system	M01: Anti-inflammatory and antirheumatic drugs	Diclofenac
		M02–M05, M09†	
N	Nervous system	N01: Anesthetics	Lidocaine
		N02: Analgesics	Morphine
		N03: Antiepileptics	Carbamazepine
		N04: Antiparkinson drugs	Carbidopa levodopa
		N05: Psycholeptics	Zopiclone
		N06: Psychoanaleptics	Sertraline
		N07: Other nervous system drugs	Disulfiram
P	Antiparasitic products, insecticides, and repellents	P01: Antiprotozoal drugs	Metronidazole
		P02: Anthelmintics	Mebendazole
		P03†	
R	Respiratory system	R01: Nasal preparations	Mometasone
		R03: Drugs for obstructive airway diseases	Terbutaline
		R05: Cough and cold drugs	Acetylcysteine
		R06: Antihistamines for systemic use	Alimemazine
		R07: Other respiratory system products	Nitric oxide
		R02†	
S	Sensory organs	S01: Ophthalmologicals	Brimonidine
		S02–S03†	
V	Various	V01,V03,V04,V06–V10†; V20‡	

† Codes that were not available in the Prescribed Drug Register.

‡ Codes for medications not used in Sweden.

Table S2. Patterns of ADHD medication prescriptions among adults in 2013, stratified by age group and sex.

	**Male**			**Female**		
	**Young adults**[†] (N=11,220)	**Middle-aged adults** (N=8,931)	**Older adults** (N=2,339)	**Young adults** (N=9,409)	**Middle-aged adults** (N=7,958)	**Older adults** (N=1,983)
**Type (%)**						
Methylphenidate	9,951 (88.69%)	7,759 (86.88%)	1,956 (83.63%)	8,430 (89.60%)	6,913 (86.87%)	1,628 (82.10%)
Dexamfetamine	237 (2.11%)	529 (5.92%)	145 (6.20%)	205 (2.18%)	416 (5.23%)	109 (5.50%)
Amfetamine	78 (0.70%)	141 (1.58%)	87 (3.72%)	63 (0.67%)	196 (2.46%)	131 (6.61%)
Atomoxetine	1,768 (15.76%)	1,165 (13.04%)	307 (13.13%)	1,468 (15.60%)	1,067 (13.41%)	240 (12.10%)
Lisdexamfetamine	92 (0.82%)	104 (1.16%)	19 (0.81%)	45 (0.48%)	73 (0.92%)	14 (0.71%)
Guanfacine	7 (0.06%)	1 (0.01%)	1 (0.04%)	0 (0%)	0 (0%)	0 (0%)
**Source (%)**						
Primary care	180 (1.60%)	96 (1.07%)	42 (1.80%)	100 (1.06%)	49 (0.62%)	27 (1.36%)
Specialist care [‡]	821 (7.32%)	538 (6.02%)	218 (9.32%)	572 (6.08%)	527 (6.62%)	236 (11.90%)
Psychiatric care	10,219 (91.08%)	8,297 (92.90%)	2,079 (88.88%)	8,737 (92.86%)	7,382 (92.76%)	1,720 (86.74%)
**Duration of medication (%)**						
Single prescription [§]	865 (7.71%)	345 (3.86%)	99 (4.23%)	573 (6.09%)	341 (4.28%)	98 (4.94%)
Short term (≤ 6m)	1,207 (10.76%)	645 (7.22%)	146 (6.24%)	982 (10.44%)	519 (6.52%)	127 (6.40%)
Medium term (6-12m)	1,285 (11.45%)	771 (8.63%)	166 (7.10%)	1,149 (12.21%)	653 (8.21%)	144 (7.26%)
Long term (> 12m)	7,863 (70.08%)	7,170 (80.28%)	1,928 (82.43%)	6,705 (71.26%)	6,445 (80.99%)	1,614 (81.39%)

[†] Young adults refer to patients aged 18-29 years, middle-aged adults refers to patients aged 30-49 years, and older adults referred to patients aged 50-64 years.
[‡] Specialist care excluding psychiatry.
[§] Single prescription here entail single dispensed prescriptions of any ADHD medication. Switching between different types of ADHD medications is not captured by this number.

Table S3. The ten most commonly dispensed respiratory system medications among individuals receiving ADHD medications by age category.

Name	ATC	Frequency	Percentage
Young adults (18-29 years)			
Alimemazine	R06AD01	2,424	11.75%
Promethazine	R06AD02	2,078	10.07%
Mometasone	R01AD09	1,628	7.89%
Opium derivatives and expectorants	R05FA02	1,528	7.41%
Terbutaline	R03AC03	1,450	7.03%
Combinations	R05CB10	1,145	5.55%
Desloratadine	R06AX27	1,112	5.39%
Budesonide	R03BA02	1,000	4.85%
Salbutamol	R03AC02	727	3.52%
Loratadine	R06AX13	616	2.99%
Middle-aged adults (30-49 years)			
Alimemazine	R06AD01	2,799	16.57%
Opium derivatives and expectorants	R05FA02	2,164	12.81%
Promethazine	R06AD02	2,065	12.23%
Mometasone	R01AD09	2,014	11.92%
Terbutaline	R03AC03	1,692	10.02%
Budesonide	R03BA02	1,249	7.40%
Combinations	R05CB10	1,098	6.50%
Desloratadine	R06AX27	1,063	6.29%
Salbutamol	R03AC02	873	5.17%
Acetylcysteine	R05CB01	831	4.92%
Older adults (50-64 years)			
Alimemazine	R06AD01	692	16.01%
Opium derivatives and expectorants	R05FA02	586	13.56%
Mometasone	R01AD09	532	12.31%
Acetylcysteine	R05CB01	461	10.67%
Promethazine	R06AD02	411	9.51%
Terbutaline	R03AC03	403	9.32%
Combinations	R05CB10	319	7.38%
Formoterol and budesonide	R03AK07	315	7.29%
Budesonide	R03BA02	305	7.06%
Salbutamol	R03AC02	292	6.76%

Table S4. The ten most commonly dispensed alimentary tract and metabolic medications among individuals receiving ADHD medications by age category.

Name	ATC	Frequency	Percentage
Young adults (18-29 years)			
Omeprazole	A02BC01	1,951	9.46%
Sodium fluoride	A01AA01	1,214	5.88%
Combinations of sodium fluoride and sodium monofluorophosphate	A01AA30	561	2.72%
Esomeprazole	A02BC05	225	1.09%
Insulin aspart	A10AB05	173	0.84%
Loperamide	A07DA03	167	0.81%
Insulin glargine	A10AE04	136	0.66%
Ranitidine	A02BA02	128	0.62%
Benzydamine	A01AD02	127	0.62%
Nystatin	A07AA02	119	0.58%
Middle-aged adults (30-49 years)			
Omeprazole	A02BC01	3,169	18.76%
Sodium fluoride	A01AA01	1,410	8.35%
Combinations of sodium fluoride and sodium monofluorophosphate	A01AA30	989	5.86%
Esomeprazole	A02BC05	468	2.77%
Metformin	A10BA02	328	1.94%
Loperamide	A07DA03	310	1.84%
Nystatin	A07AA02	224	1.33%
Insulin aspart	A10AB05	145	0.86%
Ranitidine	A02BA02	142	0.84%
Insulin glargine	A10AE04	140	0.83%
Older adults (50-64 years)			
Omeprazole	A02BC01	1,238	28.64%
Sodium fluoride	A01AA01	463	10.71%
Combinations of sodium fluoride and sodium monofluorophosphate	A01AA30	324	7.50%
Metformin	A10BA02	216	5.00%
Esomeprazole	A02BC05	191	4.42%
Loperamide	A07DA03	148	3.42%
Nystatin	A07AA02	120	2.78%
Ranitidine	A02BA02	71	1.64%
Insulin (human)	A10AC01	68	1.57%
Insulin aspart	A10AB05	54	1.25%

Table S5. The ten most commonly dispensed cardiovascular system medications among individuals receiving ADHD medications by age category.

Name	ATC	Frequency	Percentage
Young adults (18-29 years)			
Propranolol	C07AA05	615	2.98%
Epinephrine	C01CA24	243	1.18%
Metoprolol	C07AB02	241	1.17%
Hydrocortisone	C05AA01	195	0.95%
Prednisolone	C05AA04	178	0.86%
Enalapril	C09AA02	71	0.34%
Bisoprolol	C07AB07	64	0.31%
Atenolol	C07AB03	57	0.28%
Furosemide	C03CA01	55	0.27%
Glyceryl trinitrate	C05AE01	36	0.17%
Middle-aged adults (30-49 years)			
Propranolol	C07AA05	871	5.16%
Metoprolol	C07AB02	710	4.20%
Enalapril	C09AA02	638	3.78%
Amlodipine	C08CA01	417	2.47%
Furosemide	C03CA01	374	2.21%
Simvastatin	C10AA01	359	2.13%
Losartan	C09CA01	284	1.68%
Prednisolone	C05AA04	240	1.42%
Hydrocortisone	C05AA01	214	1.27%
Epinephrine	C01CA24	203	1.20%
Older adults (50-64 years)			
Enalapril	C09AA02	484	11.20%
Metoprolol	C07AB02	425	9.83%
Simvastatin	C10AA01	385	8.91%
Amlodipine	C08CA01	329	7.61%
Propranolol	C07AA05	271	6.27%
Furosemide	C03CA01	239	5.53%
Losartan	C09CA01	211	4.88%
Atorvastatin	C10AA05	143	3.31%
Felodipine	C08CA02	135	3.12%
Candesartan	C09CA06	131	3.03%

Table S6. The prescription of antipsychotics among individuals receiving ADHD medications by age category.

Name	ATC	Frequency	Percentage
Young adults (18-29 years)			
Quetiapine	N05AH04	1,556	7.54%
Olanzapine	N05AH03	897	4.35%
Aripiprazole	N05AX12	715	3.47%
Levomepromazine	N05AA02	530	2.57%
Risperidone	N05AX08	512	2.48%
Lithium	N05AN01	341	1.65%
Chlorprothixene	N05AF03	145	0.70%
Haloperidol	N05AD01	121	0.59%
Flupentixol	N05AF01	83	0.40%
Zuclopenthixol	N05AF05	79	0.38%
Paliperidone	N05AX13	43	0.21%
Ziprasidone	N05AE04	42	0.20%
Clozapine	N05AH02	33	0.16%
Melperone	N05AD03	32	0.16%
Perphenazine	N05AB03	21	0.10%
Pimozide	N05AG02	5	0.02%
Amisulpride	N05AL05	1	<0.01%
Middle-aged adults (30-49 years)			
Quetiapine	N05AH04	1,873	11.09%
Olanzapine	N05AH03	1,158	6.86%
Aripiprazole	N05AX12	821	4.86%
Levomepromazine	N05AA02	661	3.91%
Lithium	N05AN01	571	3.38%
Risperidone	N05AX08	429	2.54%
Haloperidol	N05AD01	161	0.95%
Zuclopenthixol	N05AF05	141	0.83%
Chlorprothixene	N05AF03	132	0.78%
Flupentixol	N05AF01	117	0.69%
Paliperidone	N05AX13	72	0.43%
Clozapine	N05AH02	57	0.34%
Perphenazine	N05AB03	56	0.33%
Ziprasidone	N05AE04	50	0.30%
Melperone	N05AD03	28	0.17%
Pimozide	N05AG02	2	0.01%
Older adults (50-64 years)			
Quetiapine	N05AH04	440	10.18%
Olanzapine	N05AH03	236	5.46%
Aripiprazole	N05AX12	174	4.03%
Levomepromazine	N05AA02	150	3.47%
Lithium	N05AN01	121	2.80%
Risperidone	N05AX08	91	2.11%
Flupentixol	N05AF01	51	1.18%
Zuclopenthixol	N05AF05	47	1.09%
Haloperidol	N05AD01	39	0.90%
Chlorprothixene	N05AF03	25	0.58%
Perphenazine	N05AB03	22	0.51%
Paliperidone	N05AX13	19	0.44%
Clozapine	N05AH02	14	0.32%
Ziprasidone	N05AE04	7	0.16%
Melperone	N05AD03	6	0.14%
Pimozide	N05AG02	2	0.05%
Prochlorperazine	N05AB04	1	0.02%

Sertindole	N05AE03	1	0.02%

Table S7. The prescription of anxiolytics, hypnotics, and sedative medications among individuals receiving ADHD medications by age category.

Name	ATC	Frequency	Percentage
Young adults (18-29 years)			
Hydroxyzine	N05BB01	3,333	16.16%
Zopiclone	N05CF01	3,108	15.07%
Melatonin	N05CH01	2,977	14.43%
Propiomazine	N05CM06	2,549	12.36%
Zolpidem	N05CF02	1,905	9.23%
Oxazepam	N05BA04	1,439	6.98%
Diazepam	N05BA01	1,014	4.92%
Alprazolam	N05BA12	504	2.44%
Nitrazepam	N05CD02	234	1.13%
Buspirone	N05BE01	223	1.08%
Zaleplon	N05CF03	99	0.48%
Flunitrazepam	N05CD03	69	0.33%
Lorazepam	N05BA06	45	0.22%
Triazolam	N05CD05	45	0.22%
Clomethiazole	N05CM02	38	0.18%
Midazolam	N05CD08	22	0.11%
Clobazam	N05BA09	6	0.03%
Valerianae radix	N05CM09	2	0.01%
Middle-aged adults (30-49 years)			
Zopiclone	N05CF01	4,717	27.93%
Hydroxyzine	N05BB01	3,378	20.00%
Propiomazine	N05CM06	3,065	18.15%
Zolpidem	N05CF02	2,799	16.57%
Oxazepam	N05BA04	2,459	14.56%
Diazepam	N05BA01	1,921	11.37%
Melatonin	N05CH01	1,707	10.11%
Alprazolam	N05BA12	1,000	5.92%
Nitrazepam	N05CD02	485	2.87%
Buspirone	N05BE01	316	1.87%
Flunitrazepam	N05CD03	189	1.12%
Zaleplon	N05CF03	186	1.10%
Clomethiazole	N05CM02	64	0.38%
Lorazepam	N05BA06	51	0.30%
Triazolam	N05CD05	47	0.28%
Midazolam	N05CD08	2	0.01%
Valerianae radix	N05CM09	2	0.01%
Clobazam	N05BA09	1	0.01%
Older adults (50-64 years)			
Zopiclone	N05CF01	1,319	30.52%
Hydroxyzine	N05BB01	844	19.53%
Propiomazine	N05CM06	784	18.14%
Zolpidem	N05CF02	771	17.84%
Oxazepam	N05BA04	741	17.14%
Diazepam	N05BA01	566	13.10%
Melatonin	N05CH01	351	8.12%
Alprazolam	N05BA12	241	5.58%
Nitrazepam	N05CD02	148	3.42%
Flunitrazepam	N05CD03	86	1.99%
Buspirone	N05BE01	63	1.46%

Zaleplon	N05CF03	57	1.32%
Clomethiazole	N05CM02	19	0.44%
Triazolam	N05CD05	15	0.35%
Lorazepam	N05BA06	14	0.32%
Midazolam	N05CD08	2	0.05%

Table S8. The prescription of antiepileptics among individuals receiving ADHD medications by age category.

Name	ATC	Frequency	Percentage
Young adults (18-29 years)			
Lamotrigine	N03AX09	1,318	6.39%
Pregabalin	N03AX16	927	4.49%
Valproic acid	N03AG01	501	2.43%
Gabapentin	N03AX12	223	1.08%
Carbamazepine	N03AF01	218	1.06%
Clonazepam	N03AE01	170	0.82%
Topiramate	N03AX11	122	0.59%
Levetiracetam	N03AX14	54	0.26%
Oxcarbazepine	N03AF02	18	0.09%
Zonisamide	N03AX15	10	0.05%
Lacosamide	N03AX18	7	0.03%
Phenobarbital	N03AA02	3	0.01%
Ethosuximide	N03AD01	3	0.01%
Sultiame	N03AX03	3	0.01%
Phenytoin	N03AB02	1	<0.01%
Rufinamide	N03AF03	1	<0.01%
Vigabatrin	N03AG04	1	<0.01%
Perampanel	N03AX22	1	<0.01%
Middle-aged adults (30-49 years)			
Lamotrigine	N03AX09	1,596	9.45%
Pregabalin	N03AX16	1,539	9.11%
Valproic acid	N03AG01	645	3.82%
Gabapentin	N03AX12	473	2.80%
Clonazepam	N03AE01	336	1.99%
Carbamazepine	N03AF01	305	1.81%
Topiramate	N03AX11	175	1.04%
Levetiracetam	N03AX14	54	0.32%
Oxcarbazepine	N03AF02	11	0.07%
Phenobarbital	N03AA02	6	0.04%
Phenytoin	N03AB02	6	0.04%
Primidone	N03AA03	2	0.01%
Zonisamide	N03AX15	2	0.01%
Rufinamide	N03AF03	1	0.01%
Vigabatrin	N03AG04	1	0.01%
Lacosamide	N03AX18	1	0.01%
Perampanel	N03AX22	1	0.01%
Older adults (50-64 years)			
Pregabalin	N03AX16	396	9.16%
Lamotrigine	N03AX09	362	8.38%
Gabapentin	N03AX12	136	3.15%
Valproic acid	N03AG01	127	2.94%
Clonazepam	N03AE01	94	2.17%
Carbamazepine	N03AF01	92	2.13%
Topiramate	N03AX11	43	0.99%
Levetiracetam	N03AX14	14	0.32%

Phenytoin	N03AB02	4	0.09%
Oxcarbazepine	N03AF02	3	0.07%
Phenobarbital	N03AA02	2	0.05%
Primidone	N03AA03	1	0.02%
Perampanel	N03AX22	1	0.02%

Table S9. The prescription of antidepressants among individuals receiving ADHD medications by age category.

Name	ATC	Frequency	Percentage
Young adults (18-29 years)			
Sertraline	N06AB06	2,794	13.54%
Mirtazapine	N06AX11	1,617	7.84%
Fluoxetine	N06AB03	1,457	7.06%
Escitalopram	N06AB10	1,167	5.66%
Bupropion	N06AX12	1,131	5.48%
Venlafaxine	N06AX16	1,111	5.39%
Citalopram	N06AB04	868	4.21%
Duloxetine	N06AX21	572	2.77%
Agomelatine	N06AX22	383	1.86%
Amitriptyline	N06AA09	221	1.07%
Paroxetine	N06AB05	218	1.06%
Clomipramine	N06AA04	148	0.72%
Mianserin	N06AX03	131	0.64%
Reboxetine	N06AX18	76	0.37%
Moclobemide	N06AG02	16	0.08%
Nortriptyline	N06AA10	6	0.03%
Fluvoxamine	N06AB08	3	0.01%
Tranylcypromine	N06AF04	2	0.01%
Maprotiline	N06AA21	1	<0.01%
Tryptophan	N06AX02	1	<0.01%
Middle-aged adults (30-49 years)			
Sertraline	N06AB06	2,244	13.29%
Mirtazapine	N06AX11	1,849	10.95%
Venlafaxine	N06AX16	1,668	9.88%
Escitalopram	N06AB10	1,602	9.49%
Bupropion	N06AX12	1,599	9.47%
Citalopram	N06AB04	1,231	7.29%
Fluoxetine	N06AB03	1,198	7.09%
Duloxetine	N06AX21	1,077	6.38%
Agomelatine	N06AX22	507	3.00%
Amitriptyline	N06AA09	478	2.83%
Paroxetine	N06AB05	350	2.07%
Clomipramine	N06AA04	272	1.61%
Mianserin	N06AX03	201	1.19%
Reboxetine	N06AX18	169	1.00%
Moclobemide	N06AG02	32	0.19%
Nortriptyline	N06AA10	19	0.11%
Fluvoxamine	N06AB08	6	0.04%
Tranylcypromine	N06AF04	4	0.02%
Tryptophan	N06AX02	4	0.02%
Trimipramine	N06AA06	2	0.01%
Desvenlafaxine	N06AX23	2	0.01%
Lofepramine	N06AA07	1	0.01%
Maprotiline	N06AA21	1	0.01%

Phenelzine	N06AF03	1	0.01%
Older adults (50-64 years)			
Mirtazapine	N06AX11	507	11.73%
Sertraline	N06AB06	464	10.74%
Bupropion	N06AX12	453	10.48%
Venlafaxine	N06AX16	407	9.42%
Escitalopram	N06AB10	371	8.58%
Citalopram	N06AB04	363	8.40%
Duloxetine	N06AX21	330	7.64%
Fluoxetine	N06AB03	258	5.97%
Amitriptyline	N06AA09	190	4.40%
Agomelatine	N06AX22	125	2.89%
Paroxetine	N06AB05	103	2.38%
Clomipramine	N06AA04	96	2.22%
Mianserin	N06AX03	72	1.67%
Reboxetine	N06AX18	51	1.18%
Moclobemide	N06AG02	11	0.25%
Nortriptyline	N06AA10	9	0.21%
Tryptophan	N06AX02	2	0.05%
Trimipramine	N06AA06	1	0.02%
Fluvoxamine	N06AB08	1	0.02%
Trazodone	N06AX05	1	0.02%

Table S10. The prescription of drugs used in addictive disorders among individuals receiving ADHD medications by age category.

Name	ATC	Frequency	Percentage
Young adults (18-29 years)			
Disulfiram	N07BB01	307	1.49%
Naltrexone	N07BB04	162	0.79%
Varenicline	N07BA03	137	0.66%
Acamprosate	N07BB03	116	0.56%
Buprenorphine. combinations	N07BC51	37	0.18%
Buprenorphine	N07BC01	36	0.17%
Nicotine	N07BA01	32	0.16%
Methadone	N07BC02	30	0.15%
Nalmefene	N07BB05	3	0.01%
Drugs used in opioid dependence	N07BC	1	<0.01%
Middle-aged adults (30-49 years)			
Disulfiram	N07BB01	568	3.36%
Varenicline	N07BA03	366	2.17%
Naltrexone	N07BB04	316	1.87%
Acamprosate	N07BB03	265	1.57%
Methadone	N07BC02	165	0.98%
Buprenorphine	N07BC01	130	0.77%
Buprenorphine. combinations	N07BC51	100	0.59%
Nicotine	N07BA01	71	0.42%
Nalmefene	N07BB05	5	0.03%
Drugs used in opioid dependence	N07BC	1	0.01%
Older adults (50-64 years)			
Disulfiram	N07BB01	149	3.45%
Varenicline	N07BA03	114	2.64%
Naltrexone	N07BB04	113	2.61%
Acamprosate	N07BB03	73	1.69%
Methadone	N07BC02	46	1.06%
Nicotine	N07BA01	39	0.90%
Buprenorphine	N07BC01	32	0.74%
Buprenorphine. combinations	N07BC51	14	0.32%
Nalmefene	N07BB05	2	0.05%

Table S11. The prescription of opioids among individuals receiving ADHD medications by age category.

Name	ATC	Frequency	Percentage
Young adults (18-29 years)			
Codeine. combinations excl. psycholeptics	N02AA59	1,821	8.83%
Tramadol	N02AX02	927	4.49%
Oxycodone	N02AA05	539	2.61%
Morphine	N02AA01	139	0.67%
Morphine and antispasmodics	N02AG01	129	0.63%
Buprenorphine	N02AE01	74	0.36%
Ketobemidone	N02AB01	40	0.19%
Oxycodone and naloxone	N02AA55	25	0.12%
Fentanyl	N02AB03	13	0.06%
Tapentadol	N02AX06	11	0.05%
Ketobemidone and antispasmodics	N02AG02	5	0.02%
Middle-aged adults (30-49 years)			
Codeine. combinations excl. psycholeptics	N02AA59	2,904	17.19%
Tramadol	N02AX02	1,978	11.71%
Oxycodone	N02AA05	949	5.62%
Morphine	N02AA01	337	2.00%
Buprenorphine	N02AE01	221	1.31%
Morphine and antispasmodics	N02AG01	181	1.07%
Ketobemidone	N02AB01	116	0.69%
Oxycodone and naloxone	N02AA55	64	0.38%
Fentanyl	N02AB03	47	0.28%
Tapentadol	N02AX06	41	0.24%
Ketobemidone and antispasmodics	N02AG02	14	0.08%
Hydromorphone	N02AA03	2	0.01%
Pethidine	N02AB02	1	0.01%
Older adults (50-64 years)			
Codeine. combinations excl. psycholeptics	N02AA59	832	19.25%
Tramadol	N02AX02	611	14.14%
Oxycodone	N02AA05	291	6.73%
Morphine	N02AA01	132	3.05%
Buprenorphine	N02AE01	95	2.20%
Morphine and antispasmodics	N02AG01	45	1.04%
Ketobemidone	N02AB01	44	1.02%
Oxycodone and naloxone	N02AA55	42	0.97%
Fentanyl	N02AB03	26	0.60%
Tapentadol	N02AX06	10	0.23%
Ketobemidone and antispasmodics	N02AG02	7	0.16%
Hydromorphone	N02AA03	1	0.02%
Dextropropoxyphene	N02AC04	1	0.02%
Pentazocine	N02AD01	1	0.02%

Figure S1. Dispensation of somatic medications (according to ATC classes) among individuals diagnosed with ADHD and dispensed with ADHD medications, by age group.

Co-medication	ADHD drug users (N = 35,443)	Control group (N = 35,443)	Odds ratio (95% CI)
A Alimentary tract and metabolisma			
Young adults (18-29 years)	3,595 (19.9%)	954 (5.3%)	4.6 (4.2, 4.9)
Middle-aged adults (30-49 years)	4,775 (34.0%)	1,386 (9.9%)	4.8 (4.5, 5.1)
Older adults (50-64 years)	1,578 (47.8%)	609 (18.5%)	4.1 (3.7, 4.6)
C Cardiovascular System			
Young adults (18-29 years)	1,576 (8.7%)	382 (2.1%)	4.5 (4.0, 5.0)
Middle-aged adults (30-49 years)	3,320 (23.6%)	1,058 (7.5%)	3.9 (3.6, 4.2)
Older adults (50-64 years)	1,494 (45.3%)	899 (27.3%)	2.2 (2.0, 2.5)
D Dermatologicals			
Young adults (18-29 years)	2,263 (12.5%)	933 (5.2%)	2.6 (2.4, 2.9)
Middle-aged adults (30-49 years)	2,272 (16.2%)	836 (5.9%)	3.1 (2.8, 3.3)
Older adults (50-64 years)	717 (21.7%)	298 (9.0%)	2.8 (2.4, 3.3)
G Genitourinary system and reproductive hormones			
Young adults (18-29 years)	5,239 (29.0%)	4,120 (22.8%)	1.7 (1.6, 1.8)
Middle-aged adults (30-49 years)	3,134 (22.3%)	1,908 (13.6%)	2.0 (1.8, 2.1)
Older adults (50-64 years)	921 (27.9%)	413 (12.5%)	2.8 (2.4, 3.2)
H Systemic hormonal preparations			
Young adults (18-29 years)	1,477 (8.2%)	609 (3.4%)	2.6 (2.3, 2.8)
Middle-aged adults (30-49 years)	2,097 (14.9%)	982 (7.0%)	2.4 (2.2, 2.6)
Older adults (50-64 years)	640 (19.4%)	293 (8.9%)	2.5 (2.2, 3.0)
J Antiinfectives for systemic use			
Young adults (18-29 years)	7,268 (40.2%)	3,442 (19.0%)	3.0 (2.9, 3.2)
Middle-aged adults (30-49 years)	6,569 (46.7%)	2,753 (19.6%)	3.7 (3.5, 4.0)
Older adults (50-64 years)	1,600 (48.5%)	658 (19.9%)	3.9 (3.5, 4.3)
L Antineoplastic and immunomodulating agents			
Young adults (18-29 years)	155 (0.9%)	106 (0.6%)	1.5 (1.1, 1.9)
Middle-aged adults (30-49 years)	246 (1.8%)	196 (1.4%)	1.3 (1.0, 1.5)
Older adults (50-64 years)	98 (3.0%)	64 (1.9%)	1.5 (1.1, 2.1)
M Musculoskeletal system			
Young adults (18-29 years)	3,479 (19.2%)	1,218 (6.7%)	3.4 (3.1, 3.6)
Middle-aged adults (30-49 years)	4,860 (34.6%)	1,737 (12.4%)	3.8 (3.6, 4.0)
Older adults (50-64 years)	1,345 (40.8%)	601 (18.2%)	3.1 (2.8, 3.5)
P Antiparasitic products, insecticides and repellents			
Young adults (18-29 years)	735 (4.1%)	338 (1.9%)	2.3 (2.0, 2.6)
Middle-aged adults (30-49 years)	639 (4.5%)	249 (1.8%)	2.7 (2.3, 3.1)
Older adults (50-64 years)	149 (4.5%)	66 (2.0%)	2.3 (1.7, 3.1)
R Respiratory system			
Young adults (18-29 years)	7,766 (42.9%)	2,474 (13.7%)	4.9 (4.7, 5.2)
Middle-aged adults (30-49 years)	7,931 (56.4%)	2,601 (18.5%)	5.9 (5.6, 6.3)
Older adults (50-64 years)	1,994 (60.4%)	729 (22.1%)	5.5 (4.9, 6.1)
S Sensory organs			
Young adults (18-29 years)	1,300 (7.2%)	570 (3.2%)	2.4 (2.2, 2.6)
Middle-aged adults (30-49 years)	1,422 (10.1%)	586 (4.2%)	2.6 (2.4, 2.9)
Older adults (50-64 years)	446 (13.5%)	197 (6.0%)	2.5 (2.1, 3.0)

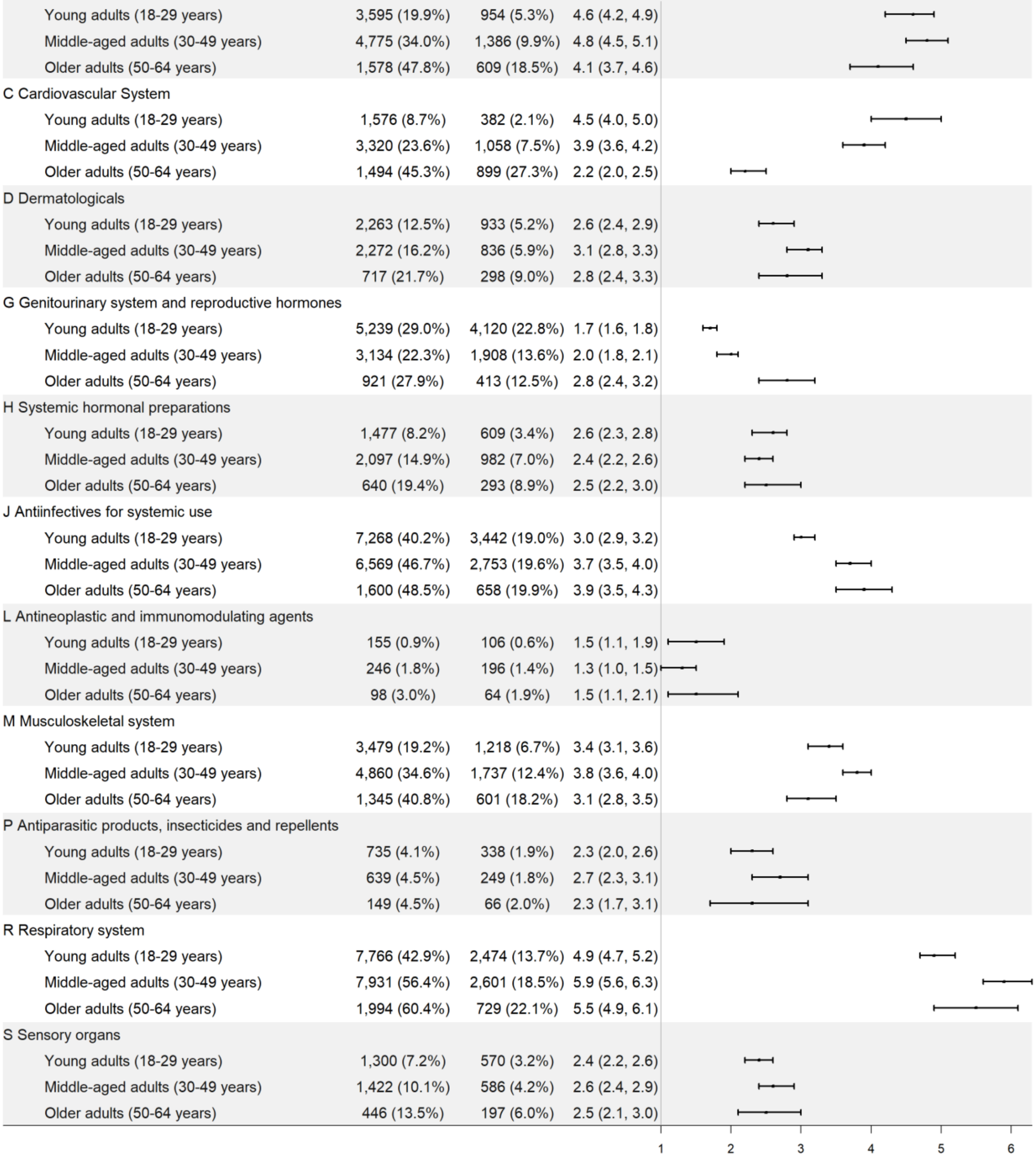

Figure S2. Dispensation of additional classes of medications (according to ATC codes) among individuals using ADHD medications, stratified by sex.

Co-medication	Sex	ADHD drug users (N = 41,840)	Control group (N = 41,840)	Odds ratio (95% CI)
A Alimentary tract and metabolisma	Male	5,275 (23.5%)	1,650 (7.3%)	4.1 (3.9, 4.4)
	Female	6,538 (33.8%)	1,907 (9.9%)	4.9 (4.6, 5.2)
C Cardiovascular System	Male	3,829 (17.0%)	1,548 (6.9%)	3.2 (3.0, 3.4)
	Female	3,805 (19.7%)	1,353 (7.0%)	3.6 (3.4, 3.9)
D Dermatologicals	Male	2,603 (11.6%)	1,119 (5.0%)	2.5 (2.3, 2.7)
	Female	3,675 (19.0%)	1,352 (7.0%)	3.1 (2.9, 3.4)
G Genitourinary system and reproductive hormones	Male	1,790 (8.0%)	412 (1.8%)	5.0 (4.5, 5.6)
	Female	9,237 (47.7%)	7,103 (36.7%)	1.6 (1.6, 1.7)
H Systemic hormonal preparations	Male	1,746 (7.8%)	675 (3.0%)	2.7 (2.5, 3.0)
	Female	3,391 (17.5%)	1,605 (8.3%)	2.4 (2.2, 2.6)
J Antiinfectives for systemic use	Male	7,534 (33.5%)	3,133 (13.9%)	3.1 (3.0, 3.3)
	Female	10,689 (55.2%)	4,968 (25.7%)	3.6 (3.4, 3.7)
L Antineoplastic and immunomodulating agents	Male	300 (1.3%)	180 (0.8%)	1.7 (1.4, 2.0)
	Female	365 (1.9%)	259 (1.3%)	1.4 (1.2, 1.7)
M Musculoskeletal system	Male	5,145 (22.9%)	1,983 (8.8%)	3.2 (3.0, 3.4)
	Female	6,298 (32.5%)	2,301 (11.9%)	3.7 (3.5, 3.9)
P Antiparasitic products, insecticides and repellents	Male	442 (2.0%)	245 (1.1%)	1.8 (1.6, 2.1)
	Female	1,360 (7.0%)	525 (2.7%)	2.7 (2.5, 3.0)
R Respiratory system	Male	9,574 (42.6%)	2,967 (13.2%)	5.0 (4.8, 5.2)
	Female	11,047 (57.1%)	3,998 (20.7%)	5.2 (5.0, 5.5)
S Sensory organs	Male	1,686 (7.5%)	769 (3.4%)	2.3 (2.1, 2.5)
	Female	2,153 (11.1%)	873 (4.5%)	2.7 (2.5, 2.9)

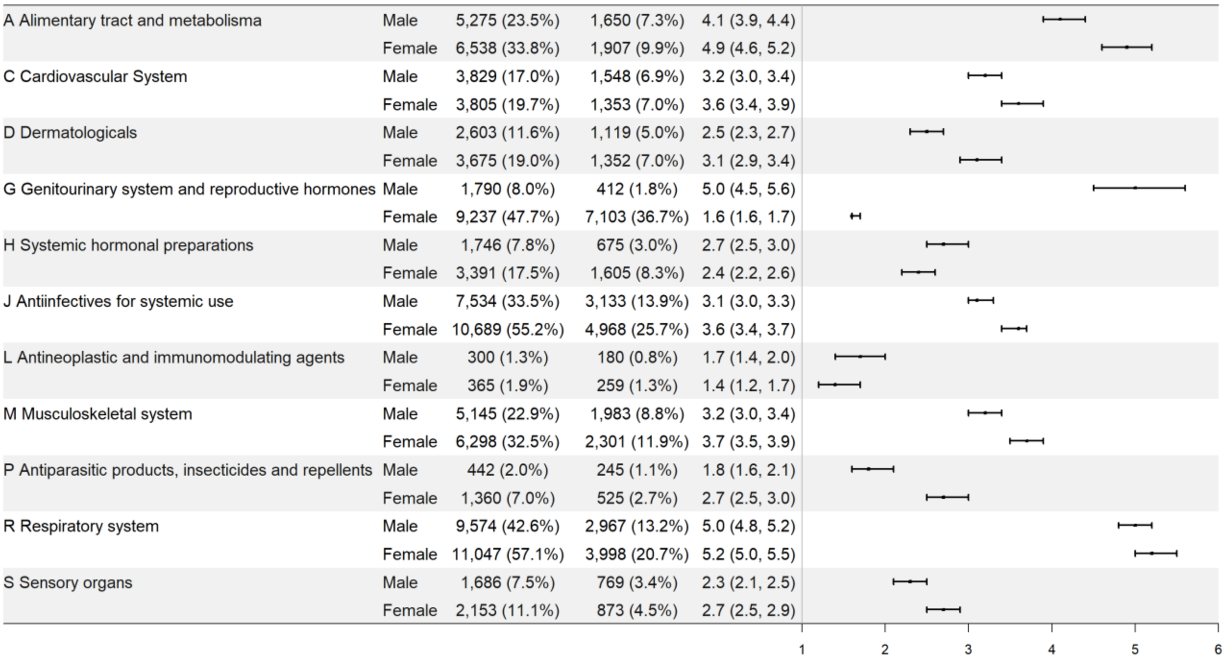

Figure S3. Dispensation of common psychotropic medications among individuals diagnosed with ADHD and dispensed with ADHD medications, by age group.

Co-medication	ADHD drug users (N = 35,443)	Control group (N = 35,443)	Odds ratio (95% CI)
Antidepressants			
Young adults (18-29 years)	7,138 (39.5%)	864 (4.8%)	14.2 (13.2, 15.3)
Middle-aged adults (30-49 years)	7,920 (56.4%)	1,271 (9.0%)	13.6 (12.7, 14.6)
Older adults (50-64 years)	1,901 (57.6%)	335 (10.2%)	12.7 (11.1, 14.5)
Antiepileptics			
Young adults (18-29 years)	2,495 (13.8%)	190 (1.1%)	15.6 (13.5, 18.1)
Middle-aged adults (30-49 years)	3,441 (24.5%)	285 (2.0%)	15.7 (13.9, 17.8)
Older adults (50-64 years)	787 (23.9%)	101 (3.1%)	10.0 (8.1, 12.4)
Antipsychotics			
Young adults (18-29 years)	2,967 (16.4%)	155 (0.9%)	23.4 (19.8, 27.5)
Middle-aged adults (30-49 years)	3,498 (24.9%)	170 (1.2%)	27.1 (23.2, 31.7)
Older adults (50-64 years)	787 (23.9%)	51 (1.5%)	20.0 (15.0, 26.7)
Anxiolytics, hypnotics and sedatives			
Young adults (18-29 years)	8,119 (44.9%)	775 (4.3%)	19.6 (18.1, 21.2)
Middle-aged adults (30-49 years)	8,789 (62.5%)	1,092 (7.8%)	20.5 (19.1, 22.1)
Older adults (50-64 years)	2,237 (67.8%)	426 (12.9%)	14.9 (13.1, 16.9)
Drugs used in addictive disorders			
Young adults (18-29 years)	628 (3.5%)	25 (0.1%)	26.4 (17.7, 39.4)
Middle-aged adults (30-49 years)	1,486 (10.6%)	78 (0.6%)	21.3 (17.0, 26.8)
Older adults (50-64 years)	408 (12.4%)	38 (1.2%)	12.2 (8.7, 17.0)
Opioids			
Young adults (18-29 years)	2,530 (14.0%)	647 (3.6%)	4.5 (4.1, 4.9)
Middle-aged adults (30-49 years)	4,068 (28.9%)	1,020 (7.3%)	5.3 (4.9, 5.7)
Older adults (50-64 years)	1,146 (34.7%)	365 (11.1%)	4.3 (3.8, 4.9)

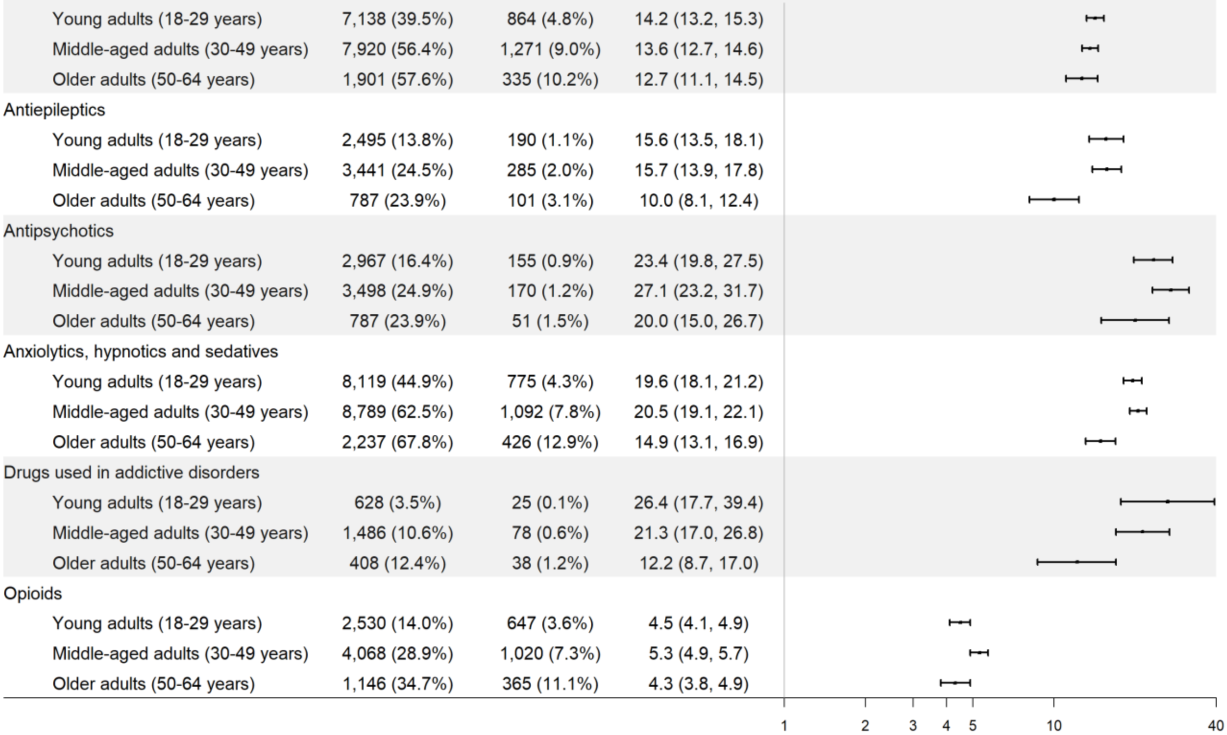

Supplement: Supplementary_material – Supplemental material for Comedication and Polypharmacy With ADHD Medications in Adults: A Swedish Nationwide Study [file Supplementary_material.pdf]
